# Temporal Expression of *Wnt1* Defines the Competency State and Terminal Identity of Progenitors in the Developing Cochlear Nucleus and Inferior Colliculus

**DOI:** 10.3389/fnana.2017.00067

**Published:** 2017-08-22

**Authors:** Stephen Brown, Mark Zervas

**Affiliations:** ^1^Department of Molecular Biology, Cell Biology and Biochemistry, Division of Biology and Medicine, Brown University, Providence RI, United States; ^2^Department of Neuroscience, Division of Biology and Medicine, Brown University, Providence RI, United States; ^3^Department of Neuroscience, Amgen, Cambridge MA, United States

**Keywords:** Genetic Inducible Fate Mapping (GIFM), *Wnt1*, cell fate, inferior colliculus, cochlear nucleus, auditory nervous system

## Abstract

The auditory system contains a diverse array of interconnected anatomical structures that mediate the perception of sound. The cochlear nucleus of the hindbrain serves as the initial site of convergence for auditory stimuli, while the inferior colliculus of the midbrain serves as an integration and relay station for all ascending auditory information. We used Genetic Inducible Fate Mapping (GIFM) to determine how the timing of *Wnt1* expression is related to the competency states of auditory neuron progenitors. We demonstrate that the *Wnt1* lineage defines progenitor pools of auditory neurons in the developing midbrain and hindbrain. The timing of *Wnt1* expression specifies unique cell types during embryogenesis and follows a mixed model encompassing a brief epoch of *de novo* expression followed by rapid and progressive lineage restriction to shape the inferior colliculus. In contrast, *Wnt1* fate mapping of the embryonic hindbrain revealed *de novo* induction of *Wnt1* in auditory hindbrain progenitors, which is related to the development of biochemically distinct neurons in the cochlear nucleus. Thus, we uncovered two modes of lineage allocation that explain the relationship between the timing of *Wnt1* expression and the development of the cochlear nucleus and the inferior colliculus. Finally, our analysis of *Wnt1^sw/sw^* mutant mice demonstrated a functional requirement of *Wnt1* for the development of auditory midbrain and hindbrain neurons. Collectively, our study provides a deeper understanding of *Wnt1* lineage allocation and function in mammalian brain development.

## Introduction

The central auditory system is comprised of several anatomically distinct brain regions that are essential for normal sound processing. The innervation at multiple anatomical levels from brainstem to midbrain, and then to subcortical and cortical areas is vital for processing auditory information (Winer and Schreiner, 2005). The cochlear nucleus of the hindbrain receives topographically organized primary inputs from the cochlea of the inner ear (Osen, 1970). Neurons within the cochlear nucleus subsequently innervate the inferior colliculus (Cant and Benson, 2003; Malmierca et al., 2005), which then processes and integrates ascending information that is ultimately relayed to auditory centers in the thalamus and cerebral cortex (Aitkin and Phillips, 1984). Interestingly, the innervation and organization of the auditory hindbrain and midbrain are largely established before the onset of hearing (Friauf and Kandler, 1990; Kandler and Friauf, 1993; Gurung and Fritzsch, 2004). These findings suggest that developmental, but perhaps not activity-dependent, mechanisms play a primary role in the functional organization of the auditory system. The functional coordination between auditory centers is dependent upon the proper allocation and distribution of specific cell types during development. Concurrent with establishing mature neuronal phenotypes within the cochlear nucleus is the differential expression of the calcium-binding proteins parvalbumin (PV), calretinin (CALR), and calbindin (CALB) (Fredrich et al., 2009). However, it is unresolved how biochemically diverse neurons in the mature auditory system are established during development.

Cell fate specification of progenitors is likely to be involved in generating the diverse array of auditory neurons and the circuits they form. Neural progenitors have distinct, yet dynamic gene expression patterns which elicit unique functions at specific developmental time points (see for example Yang et al., 2013). One gene that is required for the development of the midbrain and hindbrain is *Wnt1* (Wilkinson et al., 1987; McMahon and Bradley, 1990; McMahon et al., 1992; Ellisor et al., 2012; Yang et al., 2013). We used Genetic Inducible Fate Mapping (GIFM) (Joyner and Zervas, 2006) to mark *Wnt1*-expressing progenitors with fine temporal resolution and followed their fate and terminal identity to better understand how *Wnt1* is related to the development of auditory structures. Previous fate mapping studies have demonstrated that *Wnt1*-expressing progenitors in the lower rhombic lip (LRL) of the anterior rhombencephalon and the mesencephalon contribute to the cochlear nucleus and inferior colliculus, respectively (Zervas et al., 2004; Farago et al., 2006; Nichols and Bruce, 2006). However, an important gap in the field of auditory system development has been determining the relationship between the timing of gene expression in progenitors and the terminal fate and spatial location of mature auditory neurons. Our analysis shows that the timing of *Wnt1* expression in auditory progenitors predicts the extent, distribution, and molecular profile of mature neurons. GIFM coupled with marker analysis suggests a role for *Wnt1* in the development of auditory circuits, which link the hindbrain and midbrain. From our findings, we propose two working models based on the competence model originally proposed by Livesey and Cepko (2001), to explain *Wnt1-*based cell lineage contribution to auditory structures. It should be noted that these models (lineage restriction and *de novo* expression) are not mutually exclusive.

*Wnt1* encodes a secreted glycoprotein that plays diverse roles in development, including proliferation and cell fate decisions (Lee et al., 2004; Zervas et al., 2004; Ciani and Salinas, 2005; Brown et al., 2011; Hagan and Zervas, 2012; Dingle et al., 2016; Hagan et al., 2017). *Wnt1* is expressed in the rhombencephalon and mesencephalon, which, respectively, give rise to the hindbrain and midbrain (Wilkinson et al., 1987; Zervas et al., 2004; Farago et al., 2006). Multiple genetic strategies have uncovered functional roles of *Wnt1* during development *in vivo*: (1) Targeted deletion of *Wnt1* results in perinatal lethality and a failure of the midbrain and cerebellum to develop (McMahon and Bradley, 1990; Thomas and Capecchi, 1990); (2) In contrast, conditional gene deletion reveals distinct temporal roles for *Wnt1* in midbrain development (Yang et al., 2013); and (3) Mice homozygous for a naturally occurring hypomorphic mutation of *Wnt1*, the swaying allele (*Wnt1^sw/sw^*), live to adulthood and exhibit variability in developmental and patterning deficits of the midbrain and cerebellum (Bronson and Higgins, 1967; Lane, 1967; Thomas et al., 1991; Bally-Cuif et al., 1995; Ellisor et al., 2012). However, how the hindbrain and midbrain auditory centers are affected by the *Wnt1^sw^* allele has not been determined at a cellular level. Therefore, we used marker analysis to investigate how the cochlear nucleus and inferior colliculus are organized in *Wnt1^sw/sw^* mice. We found mild to severe perturbations of the inferior colliculus and a mild, but invariant phenotype of the cochlear nucleus of *Wnt1^sw/sw^* mice. Thus, this study provides a framework to investigate divergent and common mechanisms by which *Wnt1* establishes the cochlear nucleus and inferior colliculus.

## Materials and Methods

### Mice and Genotyping

**FIGURE 1:**
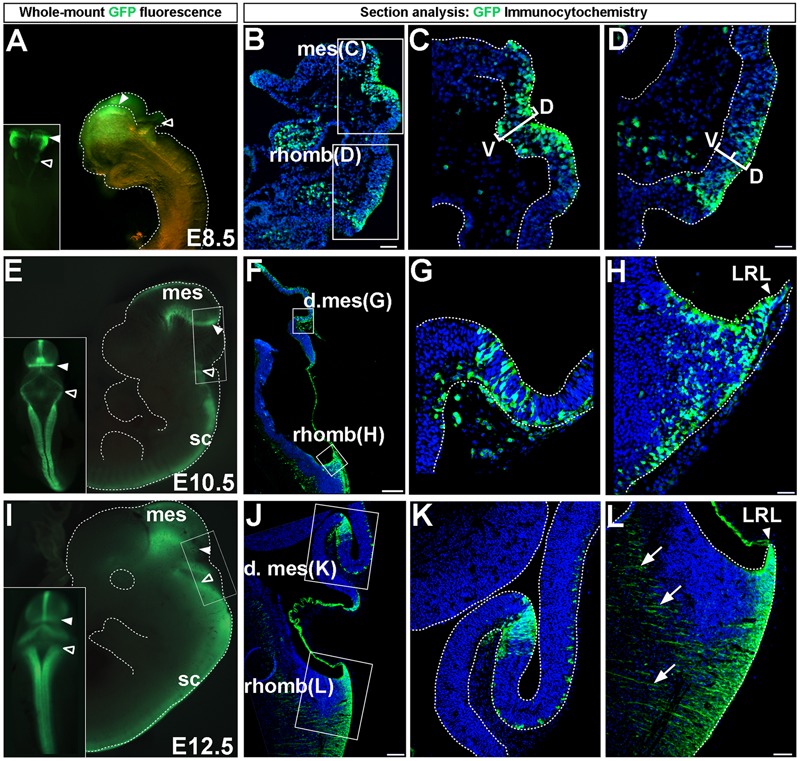
*Wnt1* expression in midbrain and hindbrain auditory primordia. *Wnt1* expression observed by GFP whole-mount fluorescence and GFP immunocytochemistry in *Wnt1-Venus* transgenic embryos. **(A–D)** Expression of *Wnt1* at E8.5. **(A)** *Wnt1* was expressed diffusely throughout the mes (solid arrowhead) and was faintly observed in the rhomb (open arrowhead). **(B–D)** Sagittal sections show detailed *Wnt1* expression within the neuroepithelium. *Wnt1* was expressed through the D-V extent of the mesencephalic neuroepithelium (**C**, bracketed area) while faint expression in the rhomb was confined to the dorsal half of the neuroepithelium (**D**, bracketed area). **(E–H)** *Wnt1* expression at E10.5. **(E)** *Wnt1* was confined to a ring at the posterior limit of the dorsal mes (solid arrowhead) while *Wnt1* in the rhomb was expressed throughout the LRL (open arrowhead). **(F)** A mid-sagittal section shows the spatial segregation of *Wnt1* expression in the mes and rhomb. **(G,H)** High magnification shows GFP positive cells remaining within the neuroepithelium. **(I–L)** *Wnt1* expression at E12.5. **(I)** The pattern of *Wnt1* expression at E12.5 was diminished within the posterior ring in the dorsal mes (solid arrowhead) and was broadened within the LRL (open arrowheads). **(J,K)** *Wnt1* in sagittal sections of the mes was restricted at E12.5 compared to the broader profile at E10.5. **(L)** The LRL showed broadened *Wnt1* expression in the neuroepithelium at E12.5. We took advantage of the likely perdurance of GFP, which allowed the early tracking of precursors as they migrate out of LRL and began to form projections (arrows). Insets in **(A,E,I)** display whole-mount dorsal views. mes, mesencephalon; d. mes, dorsal mesencephalon; rhomb, rhombencephalon; sc, spinal cord; LRL, lower rhombic lip; D-V, dorsal-ventral. Scale bars: **(B)** 63 μm; **(C,D)** 32 μm; **(F)** 260 μm; **(G,H)** 32 μm; **(J)** 130 μm; **(K,L)** 63 μm.

*Wnt1-Venus* mice were generated by C. Bromleigh and gratefully obtained from A. Joyner (Memorial Sloan Kettering Cancer Center). This line was generated by subcloning a cassette encoding yellow fluorescent protein (*Venus*, a green fluorescent protein variant) into a multi-cloning site between the translated and untranslated region of exon 1, which places *Venus* under the control of *Wnt1* regulatory elements. This same configuration was also used to control *CreER^T^* expression in *Wnt1-CreER^T^* transgenic mice (Zervas et al., 2004). The fidelity of this transgene has been verified by *in situ* hybridization (Brown et al., 2011). Our previously published studies have validated that the *Wnt1-GFP* and *Wnt1-CreER^T^* lines used in this study recapitulate endogenous *Wnt1* expression in both the midbrain and hindbrain. See for example: (1) Zervas et al. (2004) and (2) Brown et al. (2011), which show that *Wnt1-YFP* and *Wnt1-CreER*^*T*^ mimics endogenous *Wnt1* expression in the midbrain by whole mount labeling and see also (3) Supplementary Figure 1 of Ellisor et al. (2009) for comparison/validation between *Wnt1-CreER*^*T*^ and *Wnt1* in sagittal sections] and (4) Supplementary Figure 1 of Hagan and Zervas (2012) for comparison/validation between *Wnt1-Venus* and *Wnt1* in sagittal sections and Supplementary Figure 2 of Hagan and Zervas (2012) for comparison/validation between *Wnt1-CreER*^*T*^ and *Wnt1* in sagittal sections. Reporter *mGFP* mice (*Tau^lox-STOP-loxmGFP-IRES-NLS-LacZ-pA^*, Hippenmeyer et al., 2005) were generously provided by S. Arber, which allowed us to follow the *Wnt1* lineage *in vivo* (For example see: Brown et al., 2009; Ellisor et al., 2009; Ellisor and Zervas, 2010; Hagan and Zervas, 2012). Mice were housed and handled in accordance with Brown University Institutional Animal Care and Use Committee guidelines. Genotyping was done as previously described (Brown et al., 2009; Ellisor et al., 2009). *Wnt1-Venus* embryos were detected by whole-mount fluorescence (**Figure 1**) using filters for GFP as previously shown (Ellisor et al., 2012). In tissue sections, the *Wnt1-Venus* transgene was detected with anti-GFP antibodies (described below).

### Embryonic Tissue Preparation

*Wnt1-Venus* transgenic males were bred with Swiss Webster females (Taconic) to obtain litters at three different embryonic stages: E8.5, E10.5, and E12.5 (**Figure 1**). Embryos were dissected in PBS over ice and *Wnt1-Venus* embryos were identified by GFP fluorescence, imaged, and confirmed by genotyping for GFP (Ellisor et al., 2009). Embryos were fixed in 4% paraformaldehyde (PFA) overnight at 4°C, cryoprotected, and embedded in OCT. Embryos (*n* > 3 across two litters for each embryonic stage) were sectioned sagittally (12 μm) with a Leica cryostat and stored in ziplock bags at -20°C. Sections were immunolabeled as described below.

### Genetic Inducible Fate Mapping (GIFM)

**FIGURE 2:**
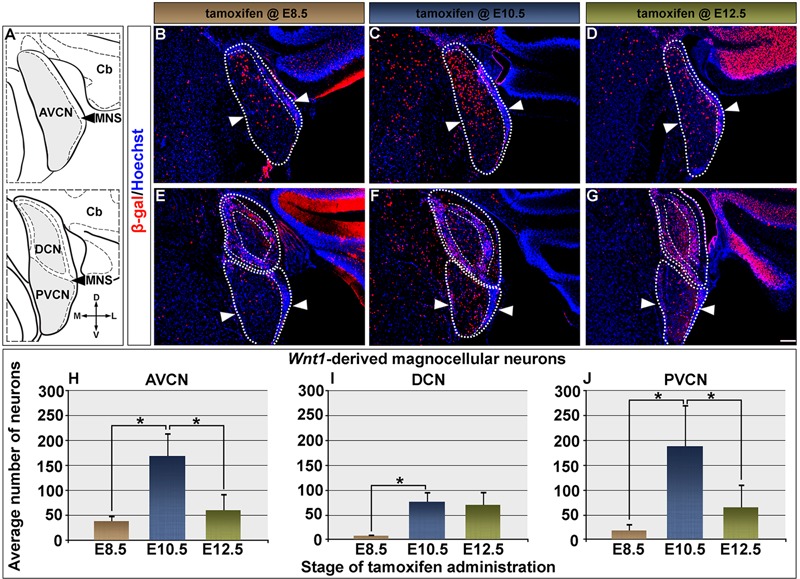
Timing of *Wnt1* expression in predicts the extent and distribution of neurons within the cochlear nucleus. **(A)** Schematic representation of the AVCN, DCN, and PVCN in coronal planes. Areas shaded in gray delineate the magnocellular core (MGC) of each nucleus, which was counted. Fine dashed lines represent the borders of the microneuronal shell (MNS); the laterally located cerebellum is denoted as Cb. *Wnt1*-derived neurons labeled by immunocytochemistry in the rest of the figure were nuclear β-gal+ (red). **(B–D)** The contribution of *Wnt1*-expressing progenitors to the AVCN are show: **(B,H)** Progenitors marked at E8.5 were predominantly found in a dorsal domain of the AVCN (above the line delineated by arrowheads) and yielded 38.9 ± 12.0 *Wnt1*-derived neurons/section. **(C,H)** *Wnt1-*expressing progenitors marked at E10.5 were dorsally distributed in the mature AVCN and gave rise to 169.4 ± 47.2 *Wnt1*-derived neurons/section. **(D,H)** By E12.5, *Wnt1*-expressing progenitors ultimately contributed to the AVCN in decreased numbers (60.8 ± 34.4 *Wnt1*-derived neurons/section). **(E–G)** Magnocellular neurons of the DCN and PVCN were derived from progenitors with late *Wnt1* expression. **(E,I)** DCN progenitors which expressed *Wnt1* at E8.5 largely contributed to the MNS, with few *Wnt1*-derived MGC neurons (8.0 ± 4.1 *Wnt1*-derived neurons/section). **(F,I)** *Wnt1*-expressing progenitors marked at E10.5 gave rise to DCN neurons throughout the D-V axis of the MGC and continued to populate the MNS (75.9 ± 19.9 *Wnt1*-derived neurons/section). **(G,I)** The contribution of *Wnt1*-expressing progenitors to DCN MGC neurons continued through E12.5 (70.2 ± 27.8 *Wnt1*-derived neurons/section). **(E–G)** *Wnt1* derived neurons populated the PVCN with a similar profile as the AVCN. **(E,J)** *Wnt1*-expressing progenitors marked at E8.5 contributed sparsely to the dorsal aspects of the PVCN (18.3 ± 14.4). **(F,J)** Peak contribution of *Wnt1*-expressing progenitors (188.4 ± 85.0) to the PVCN arose from E10.5 expression. **(G,J)** In contrast, *Wnt1*-expressing progenitors marked at E12.5 (65.1 + 46.4) contributed in fewer numbers throughout the D-V axis. Asterisks denote average numbers of *Wnt1*-derived neurons, which were significantly different (*p* < 0.005). Scale bars: 130 μm **(B–G)**.

**FIGURE 3:**
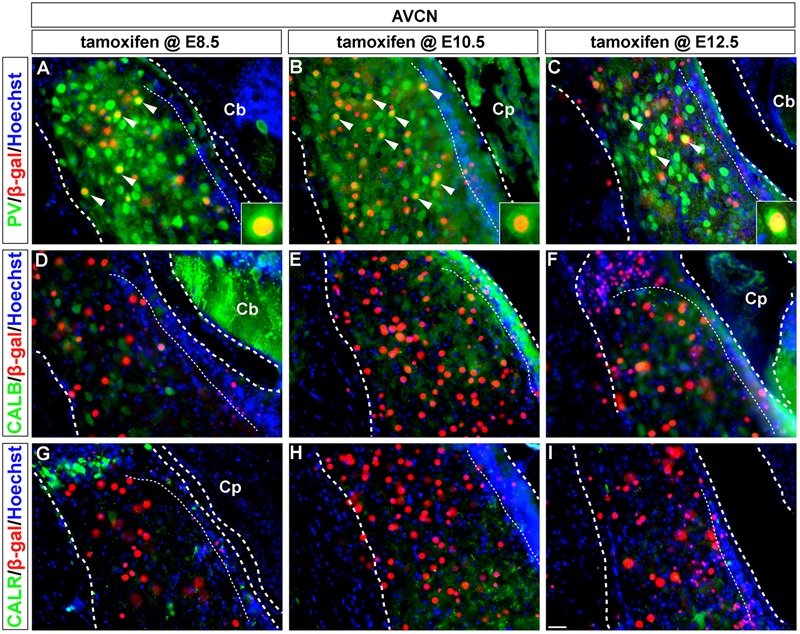
PV-expressing AVCN neurons are derived from *Wnt1*-expressing progenitors. **(A–I)** *Wnt1*-derived neurons in the AVCN were identified by nuclear β-gal immunocytochemistry (red) and with indicated biochemical markers (green). *Wnt1* marking between E8.5 and E12.5 defined biochemically distinct neurons of the AVCN, which expressed PV (**A–C**, arrowheads, insets). Neither CALB **(D–F)** nor CALR **(G–I)** expressing neurons neurons in the AVCN were derived from *Wnt1-*expressing progenitors between E8.5 and E12.5. Broad dashed lines indicate the AVCN structure, while fine dashed lines delineate the granule cell layer. Cb, cerebellum; Cp, choroid plexus. Scale bar in **(I)** indicates 32 μm.

Genetic Inducible Fate Mapping experiments were conducted by crossing *Wnt1-CreER^T^;mGFP* males with Swiss Webster wild type females (Taconic) as previously described (Brown et al., 2009; Ellisor et al., 2009; Ellisor and Zervas, 2010). The morning (0900) of the day a vaginal plug was detected was designated as 0.5 days post-coitus. Tamoxifen was administered at a dose of 4 mg to time-pregnant females by oral gavage at 0900 (Brown et al., 2009; Ellisor et al., 2009). Note that descriptions of the initially marked populations of the *Wnt1* lineage (labeled by tamoxifen administration at E8.5, E9.5, and E10.5) can be found in Zervas et al. (2004) (Figures 2B, 3B, 5E, inset, respectively). Mice were genotyped by obtaining a tail biopsy and performing PCR analysis on DNA from tail lysates. At 6 weeks of age *Wnt1-CreER^T^;mGFP* fate mapped mice were deeply anesthetized with Nembutal (100 mg/kg) and intracardially perfused with PFA. Craniotomies were performed to extract brains, which were stored in PFA at 4°C until sectioning. Brains were embedded in 3% agarose in PBS and sectioned coronally (40 μm) with a Leica vibratome (Brown et al., 2009). Fate mapped brains (*n* > 3) across two litters were processed for marking and analysis.

### Immunocytochemistry

Sections were immunolabeled as previously described (Ellisor et al., 2009). Sagittal sections from *Wnt1-Venus* embryos at E8.5, E10.5, and E12.5 were analyzed using an anti-GFP antibody (1:600, Molecular Probes, Cat # A-6455). Adult coronal sections for fate mapping experiments were immunolabeled with an anti-β-galactosidase (β-gal) antibody (1:500, Biogenesis, Cat # 4600-1409 or 1:500, Abcam, Catalog # ab9361-250) to identify *Wnt1-*derived cells (Ellisor et al., 2009). In addition, anti-Calretinin (1:5000, Chemicon; Billerica, MA; Catalog # AB1550), anti-Calbindin (1:1000, Swant, Catalog # CB3a), and anti-Parvalbumin (1:1000, Sigma, Catalog # P3088-.2ML) antibodies were used as biomarkers. Secondary antibodies were prepared at 1:500 and include: Alexa 488 (Invitrogen; Cat # A-21206, donkey anti-rabbit IgG; Cat # A-21202, donkey anti-mouse IgG; Cat #A-11055 donkey anti-goat IgG), Dylight 549 (Jackson ImmunoResearch Laboratories; Cat #703-505-155, donkey anti-chicken).

### Histology

Silver staining was performed as previously described (Gallyas, 1979; See also Zervas and Walkley, 1999). Briefly, 40 μm thick sagittal sections from *Wnt1^+/+^* and *Wnt1^sw/sw^* mice (processed in parallel) were mounted on glass slides and incubated in pyridine:acetic anhydride (2:1) for 30 min. Sections were rinsed three times in ddH_2_O for 5 min, followed by a 45 min incubation in ammoniacal silver nitrate. Sections were rinsed three times in 0.5% acetic acid for 10 min, incubated in developer solution (Gallyas, 1979) for 5 min, and terminated by incubating in 1% acetic acid for 10 min. Sections were dehydrated through ethanol followed by xylene. Slides were coverslipped with Permount mounting media.

### Microscopy and Cell Counting

Whole-mount images were obtained with a Leica MZ16F stereoscopic epifluorescent microscope using PictureFrame3 software. Images of tissue sections were obtained with a Leica DM6000B epifluorescent microscope using Volocity 5.2 imaging software (Improvision). Low magnification images were captured with 2.5× and 5× objectives while high magnification images were obtained using a motorized stage with 10× and 20× objectives. Actual magnifications are indicated in figures by scale bars. All images were pseudo colored live as part of the acquisition palettes. Imaging data sets were exported to Adobe Photoshop CS3 where montages of representative data were generated. We counted neurons in the central nucleus of the inferior colliculus by acquiring 10× images spanning the entire central nucleus that were stitched together in Adobe Photoshop CS3. Profiles in the red channel corresponded to nuclear β-gal labeling of fate mapped neurons in accordance with the genetics of our fate mapping alleles (Brown et al., 2009; Ellisor et al., 2009; Ellisor and Zervas, 2010). Images were imported into ImageJ and data was converted to binary images and counted using the Analyze Particles function. Magnocellular neurons of the cochlear nucleus were manually counted to avoid the densely labeled granule cell layers that were prohibitive to the automated method described above. Manual counting was done by analyzing images in Photoshop CS3 with the Count Tool function. For each time point, three representative sections from three separate animals (nine sections total per time point) were counted for each structure. Data are represented as the average number (± the standard deviation) of *Wnt1*-derived neurons per section. Statistical analysis using Student’s *t*-test was performed to determine the significance of change in contribution between time points of analysis.

## Results

### *Wnt1* Expression in Auditory Primordia

Prior to fate mapping the *Wnt1* lineage, we assessed the distribution of *Wnt1*-expressing progenitors in *Wnt1-Venus* embryos (Brown et al., 2011) to demonstrate where the initial populations of midbrain and hindbrain progenitors resided at key developmental stages. We evaluated GFP-labeled domains by whole-mount fluorescence and GFP immunocytochemistry at embryonic day (E)8.5, E10.5, and E12.5 (**Figure 1**). The expression patterns described below for the *Wnt1-Venus* transgenic line reflects *Wnt1* expression as detected by *in situ* hybridization (See Materials and Methods, Mice and Genotyping for details and references). *Wnt1* delineated the mesencephalon and was faintly observed in more posteriorly located rhombomeres at E8.5 (**Figure 1A**) consistent with *in situ* hybridization experiments (Wilkinson et al., 1987; Zervas et al., 2004). We also analyzed mid-sagittal sections to provide a more detailed description of *Wnt1* at E8.5 (**Figures 1B–D**). *Wnt1* spanned the dorsal-ventral (D-V) extent of the mesencephalic neuroepithelium (**Figures 1B,C**, bracketed area). However, *Wnt1* was heterogeneously expressed and progenitors not expressing *Wnt1* were also observed within the mesencephalon expression domain (**Figure 1C**). *Wnt1* in the rhombencephalon was restricted to the dorsal tier of the neuroepithelium (**Figures 1B,D**, bracketed area). At E10.5, whole mount fluorescence revealed that *Wnt1* had become confined to the dorsal and ventral midline and to a ring of expression at the posterior limit of the dorsal mesencephalon (**Figure 1E**, solid arrowhead), in agreement with previous reports (Wilkinson et al., 1987; Gavin et al., 1990; Ellisor et al., 2009). In comparison to E8.5, *Wnt1* had expanded in the LRL at E10.5 (**Figure 1E**, open arrowhead). Analysis of *Wnt1* at E10.5, in midline sagittal sections, clarified the posterior expression in the mesencephalon (**Figures 1F,G**), as well as the broadened expression throughout the LRL (**Figures 1F,H**). *Wnt1*-expressing progenitors in the neuroepithelium of the dorsal mesencephalon at E10.5 appeared clonal and were interspersed amongst non-expressing progenitors (**Figure 1G**). Similarly, progenitors that did not express *Wnt1* were found within the LRL at E10.5 (**Figure 1H**). The general whole-mount pattern of expression observed at E10.5 persisted until E12.5 (**Figure 1I**), although the lateral aspects of the posterior ring of the mesencephalon showed diminished expression (**Figure 1I**, solid arrowheads), while expression in the LRL continued to broaden (**Figure 1l**, open arrowheads). Analysis of sagittal sections confirmed that the expression domain was tightly restricted in the posterior mesencephalon at E12.5 (**Figures 1J,K**). Expression of *Wnt1* was also observed in the LRL at E12.5 (**Figures 1J,L**) and consisted of early projections (**Figure 1L**, arrows), which contributed to the broadened expression domain, as well as cells emanating from the LRL. *Wnt1*-GFP expression at E12.5 may exhibit perdurance of GFP, which allowed us to observe precursors as they migrated out of LRL and formed projections (**Figure 1L**, arrows). Note that the possibility of GFP perdurance could confound our assessment of the precursors expressing *Wnt1* at this stage. However, we provided representative data and rendered a fair interpretation of *Wnt1*-expressing progenitors.

### The *Wnt1* Lineage Contributes to the Cochlear Nucleus in Distinct Temporal Windows

Next, we addressed how *Wnt1*-expressing progenitors in the rhombencephalon contributed to the mature auditory hindbrain using GIFM (Joyner and Zervas, 2006). Specifically, we used *Wnt1-CreER^T^;mGFP* transgenic mice bred to *Swiss Webster* females to determine how the timing of *Wnt1* expression in progenitors was related to the organization of the cochlear nucleus. We administered tamoxifen to time-pregnant females between E8.5 and E14.5 and analyzed the cochlear nucleus at 6 weeks of age. The expression of the *Wnt1-CreER^T^* transgene accurately reflect *Wnt1* expression as detected by *in situ* hybridization (See Materials and Methods, Mice and Genotyping for details). In addition, a description of the initially marked populations of the *Wnt1* lineage (labeled by tamoxifen administration at E8.5, E9.5, and E10.5) can be found in Zervas et al. (2004) (Figures 2B, 3B, 5E, inset, respectively).

**FIGURE 4:**
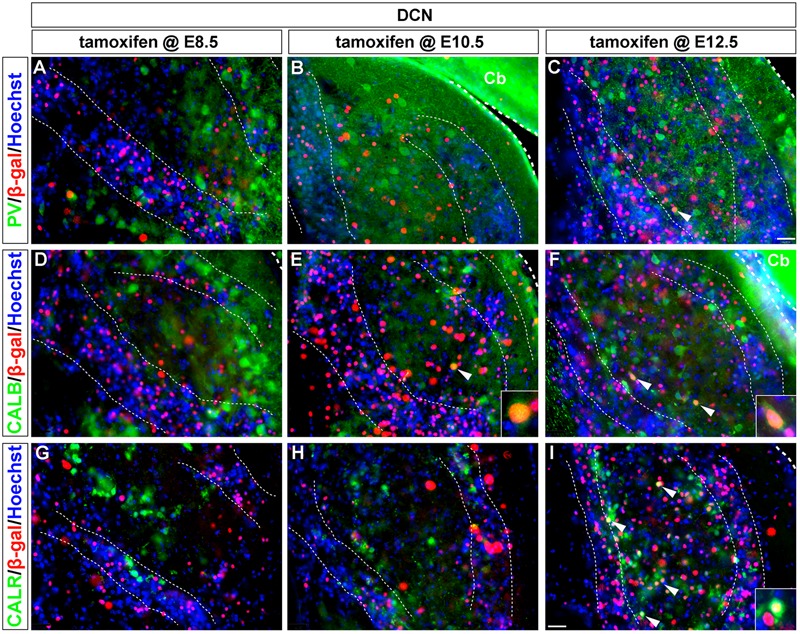
CALB and CALR-expressing DCN neurons are sequentially derived from *Wnt1-*expressing progenitors. *Wnt1*-derived neurons were identified by nuclear β-gal immunocytochemistry (red) and with biochemical markers (green). **(A–C)** PV-expressing DCN neurons were not derived from progenitors which expressed *Wnt1* between E8.5 and E12.5. **(D–F)** Progenitors expressing *Wnt1* between E10.5 and E12.5 gave rise to CALB positive neurons only when marked at E10.5 and E12.5. (arrowheads, insets). **(G–I)** CALR positive DCN neurons were derived from *Wnt1*-expressing progenitors only when marked at E12.5 (see arrowheads, inset, **I**). Broad dashed lines indicate the separation of DCN from cerebellum (Cb), while fine dashed lines depict the granule cell layers. Scale bar in **(I)** indicates 32 μm.

**FIGURE 5:**
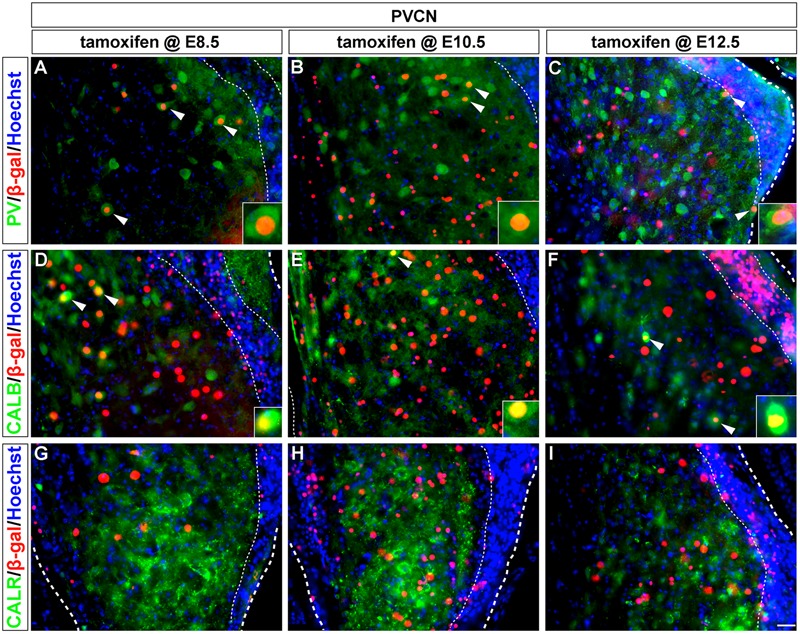
PV and CALB-expressing PVCN neurons are derived from *Wnt1*-expressing progenitors. *Wnt1*-derived neurons were identified by nuclear β-gal immunocytochemistry (red) and biochemical markers were delineated in green. PV positive (**A–C**, arrowheads, insets) and CALB positive (**D–F**, arrowheads, insets) neurons of the PVCN were descendant from progenitors that expressed *Wnt1* between E8.5 and E12.5. **(G–I)** CALR positive neurons were not derived from progenitors which expressed *Wnt1* from E8.5 to E12.5. Broad dashed lines indicate lateral or medial edge of the PVCN; fine dashed lines depict the granule cell layer. Scale bar in **(I)** indicates 32 μm.

Here, we used the *mGFP* reporter, which is driven by the *Tau* locus and allowed us to strictly assess the *Wnt1* lineage contribution to neurons (Hippenmeyer et al., 2005). Neuronal descendants of progenitors that expressed *Wnt1* (at the time of tamoxifen administration) continue to express nuclear β-galactosidase (β-gal) and were identified through immunocytochemistry (**Figures 2–5**). The hindbrain cochlear nucleus is partitioned into three distinct divisions with overlapping function and organization: the anteroventral cochlear nucleus (AVCN), dorsal cochlear nucleus (DCN), and the posteroventral cochlear nucleus (PVCN) (Ryugo and Parks, 2003). The *Wnt1* lineage contributed to neurons within subdivisions of each of the cochlear nuclei over a broad developmental window (marking from E8.5 to E12.5) (quantified in **Figure 2**). The distribution pattern of *Wnt1*-derived neurons differed amongst the individual nuclei and was dependent on the time that progenitors expressed *Wnt1*. AVCN progenitors in the LRL, which express *Wnt1* from E8.5 through E12.5 (**Figure 1**), gave rise to magnocellular neurons distributed primarily in the dorsal aspect of the AVCN with the peak contribution occurring at E10.5 (**Figures 2B–D**). However, *Wnt1*-expressing progenitors marked at E10.5 and E12.5 also contributed to neurons positioned in the ventral domain of the AVCN resulting in a more even distribution throughout the D-V axis (**Figures 2C,D**). Interestingly, granule cells within the microneuronal shell (MNS) of the AVCN were derived in greater abundance from progenitors that expressed *Wnt1* at E10.5, with the peak contribution at E12.5 (**Figures 2B–D**, compare the MNS, which is denoted in **Figure 2A**). In contrast, there was not a discernible D-V bias in the distribution of *Wnt1*-derived magnocellular neurons of the DCN at any marking stage (**Figures 2E–G**). Sparsely labeled neurons derived from *Wnt1-*expressing progenitors at E8.5 were distributed in the dorsal half of the PVCN (**Figure 2E**). *Wnt1-*expressing progenitors marked at E10.5 resulted in the peak contribution to the PVCN while progenitors marked at E12.5 contributed to neurons distributed throughout the lateral core of the PVCN and its MNS (**Figures 2F,G**).

We quantified the contribution of *Wnt1*-expressing progenitors to magnocellular neurons within the cochlear nuclei (**Figures 2H–J**). Magnocellular neurons of the AVCN were predominantly derived from progenitors expressing *Wnt1* at E10.5 (169.4 ± 47.2 *Wnt1*-derived neurons/section), which was a fourfold increase compared to marking at E8.5 (38.9 ± 12.0 *Wnt1*-derived neurons/section) (**Figure 2H**). Despite extensive *Wnt1* expression observed in the developing hindbrain at E12.5, the contribution of the *Wnt1* lineage to AVCN neurons declined to 60.8 ± 34.4 *Wnt1-*derived neurons/section (**Figure 2H**). By E14.5, progenitors expressing *Wnt1* no longer contributed to the cochlear nucleus (data not shown). In contrast to the AVCN, very few neurons within the DCN were derived from *Wnt1-*expressing progenitors marked at E8.5 (8.0 ± 4.1 *Wnt1*-derived neurons/section) (**Figure 2I**). Unlike the sharp peak of contribution observed in the AVCN when marked at E10.5, the contribution to the DCN was more limited and occurred over an extended time period (from E10.5 to E12.5) (**Figure 2I**). The number of DCN neurons derived from the *Wnt1* lineage marked at E10.5 and E12.5 were not significantly different (75.9 ± 19.9 compared to 70.2 ± 27.8 *Wnt1*-derived neurons/section, respectively) (**Figure 2I**). The contribution of *Wnt1*-expressing progenitors to PVCN neurons resembled that of the AVCN with a markedly sharp peak of contribution to the magnocellular core when marked at E10.5 (**Figure 2J**). Specifically, there was a 10-fold increase in the contribution of the *Wnt1* Lineage to the PVCN when comparing marking at E10.5 (188.4 ± 85.0 *Wnt1*-derived neurons/section) versus marking at E8.5 (18.3 ± 14.4) (**Figure 2J**). Although there was broad *Wnt1* expression in the hindbrain at E12.5, the contribution of the *Wnt1* lineage to PVCN neurons significantly decreased when marked at this stage (65.1 ± 46.4 *Wnt1*-derived neurons/section) (**Figure 2J**). In summary, *Wnt1-*expressing progenitors marked at E8.5 gave rise to neurons in cochlear nuclei with labeled neurons in the dorsal AVCN/PVCN or the MNS of the DCN. The *Wnt1* lineage marked at E10.5 resulted in the peak of contribution to AVCN/PVCN neurons. In contrast, there was prolonged, but less substantial contribution of the *Wnt1* lineage to DCN neurons from E10.5 to E12.5. Notably, the early (E8.5) expression of *Wnt1* did not produce *Wnt1-*derived neurons being evenly distributed throughout the AVCN, DCN, nor PVCN of the cochlear nucleus, which suggests that *de novo Wnt1* expression occurred in progenitors to generate the full complement of the *Wnt1* lineage contribution to the auditory hindbrain. These findings suggest that the timing of *Wnt1* is related to the specification of distinct cell types in the cochlear nucleus.

### Temporally Labeled *Wnt1*-Expressing Progenitors Contribute to Biochemically Distinct Neurons in the Cochlear Nucleus

The calcium-binding proteins PV, CALB, and CALR are expressed exclusively in neurons (Bredderman and Wasserman, 1974; Heizmann, 1984; Rogers, 1987; Celio, 1990). Moreover, the expression of PV, CALB, and CALR correlates with the functional distribution of neurons in cochlear nuclei of rat (Fredrich et al., 2009). We used these calcium-binding proteins as molecular markers to determine whether the timing of *Wnt1* expression was related to the establishment of biochemically distinct neurons of the cochlear nucleus. We did this by performing double immunocytochemistry to identify how the *Wnt1* lineage (β-gal positive, red) contributed to PV, CALB, or CALR (green) neurons in the AVCN, DCN, and PVCN (**Figures 3–5**).

Parvalbumin-positive neurons in the magnocellular core of the AVCN were derived from the *Wnt1* lineage across all stages of marking (**Figures 3A–C**, insets). These findings show that *Wnt1*-expressing progenitors contributed to the PV-positive subpopulation in the AVCN, likely inclusive of T-stellate and spherical-bushy, but not globular-bushy neurons (Pór et al., 2005; Fujiyama et al., 2009). Notably, CALB+ and CALR+ neurons in the AVCN were not derived from the *Wnt1* lineage at any marking stage (**Figures 3D–I**).

Parvalbumin-expressing neurons in the DCN, likely ML-stellate cells (Caicedo et al., 1996; Fujiyama et al., 2009), were not derived from progenitors expressing *Wnt1* at any time point examined (**Figures 4A–C**). In contrast, CALB-positive neurons in the DCN, that is fusiform and cartwheel cells (Frisina et al., 1995), were derived from progenitors expressing *Wnt1*, but only sparsely and at E10.5 and E12.5 (**Figures 4D–F**, insets). CALR-positive neurons in the DCN, likely unipolar brush cells (Floris et al., 1994; Di Bonito and Studer, 2017), were derived from the *Wnt1* Lineage, but only at E12.5 (**Figures 4G–I**).

Similar to the AVCN, *Wnt1-*expressing progenitors marked from E8.5 to E12.5 gave rise to PV-positive neurons in the PVCN, which is inclusive of T-stellate cells (Di Bonito and Studer, 2017) (**Figures 5A–C**). CALB-positive neurons of the PVCN, including putative octopus cells (Di Bonito and Studer, 2017), were also derived from the *Wnt1* lineage at each marking stage (**Figures 5D–F**), which was distinctly different compared to the AVCN. CALR+ neurons in the PVCN were not derived from the *Wnt1* lineage as any marking stage (**Figures 5G–I**). In summary, neurons expressing calcium binding proteins, which render these cohorts as functionally distinct neuronal subtypes, were derived from the *Wnt1* lineage in temporal patterns unique to each subdivision in the cochlear nucleus.

### The Auditory Midbrain Is Established through Progressive Lineage Restriction after E9.5

**FIGURE 6:**
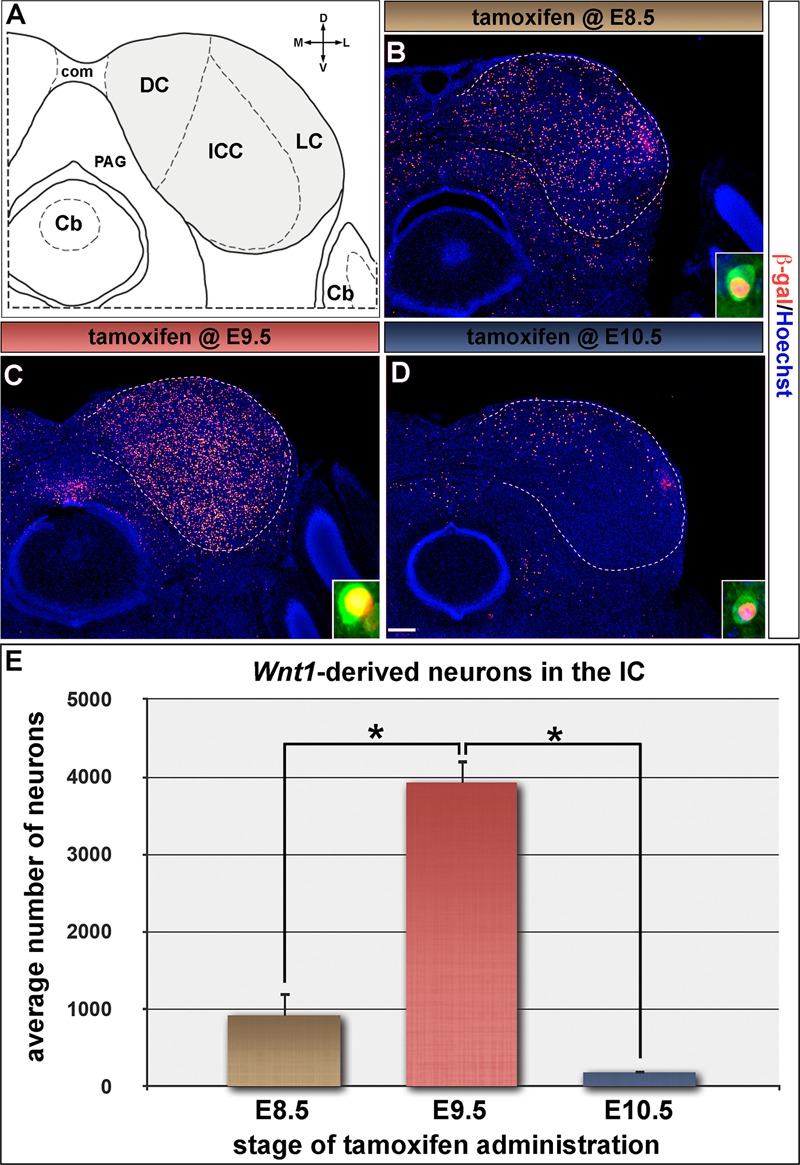
The inferior colliculus is established by progenitors expressing *Wnt1* between E8.5 and E10.5. **(A)** Schematic representation of the auditory midbrain (hemicoronal view is shown). The inferior colliculus (IC) is shaded gray to show the areas counted in **(E)**, which are partitioned by dashed lines. **(B–D)** *Wnt1*-derived neurons were identified by nuclear β-gal immunocytochemistry (red). **(B,E)** *Wnt1*-expressing progenitors marked at E8.5 gave rise to 919.3 ± 307.5 *Wnt1*-derived IC neurons/section. **(C,E)** The pool of IC neurons is largely established by *Wnt1* expressing descendants marked at E9.5 (3923.8 ± 722.7 *Wnt1*-derived IC neurons/section). **(D,E)** *Wnt1*-expressing progenitors at E10.5 contributed sparsely to IC neurons (181.7 ± 132.8 *Wnt1*-derived IC neurons/section), which largely populated the dorsomedial IC. Insets in **(B–D)** depict representative PV positive neurons of the ICC derived from *Wnt1*-expressing progenitors. Asterisks denote average numbers of *Wnt1*-derived neurons which were significantly different (*p* < 0.001). Scale bar: 260 μm **(B–D)**.

We next determined how *Wnt1*-expressing progenitors in the mesencephalon contributed to the auditory midbrain using GIFM, as described above for the cochlear nucleus. *Wnt1-*expressing progenitors at E8.5 (**Figure 1A**, solid arrowheads) were marked and assed for their contribution and distribution in the adult midbrain (schematic view shown in **Figure 6A**). Marking at E8.5 resulted in neurons being distributed throughout the inferior colliculus (**Figure 6B**). Quantitative analysis showed that progenitors marked at E8.5 gave rise to 919.3 ± 307.5 *Wnt1*-derived neurons/section (**Figure 6E**). Marking at E9.5, which is subsequent to neural tube closure, resulted in a substantial peak of labeled neurons contributing to the inferior colliculus in adulthood (**Figure 6C**). Specifically, we observed a fourfold increase in the number of marked neurons at E9.5 (3923.8 ± 722.7 *Wnt1*-derived neurons/section) compared to marking a day earlier (**Figure 6E**). The large increase in contribution indicates that *de novo* expression of *Wnt1* occurred as the mesencephalon underwent morphometric changes likely necessary for neural tube closure. Despite the preserved *Wnt1* expression domain at E10.5, there was a rapid and large decline in the lineage contribution (181.7 ± 132.8 *Wnt1*-derived neurons/section) to the inferior colliculus marked at this stage (**Figures 6D,E**). *Wnt1*-expressing progenitors contributed to PV-expressing neurons, which were found throughout each of the inferior colliculus subdivisions (Coleman et al., 1992; Vater and Braun, 1994; Lohmann and Friauf, 1996; Paloff et al., 2004; Sharma et al., 2009) (insets in **Figures 6B–D**).

**FIGURE 7:**
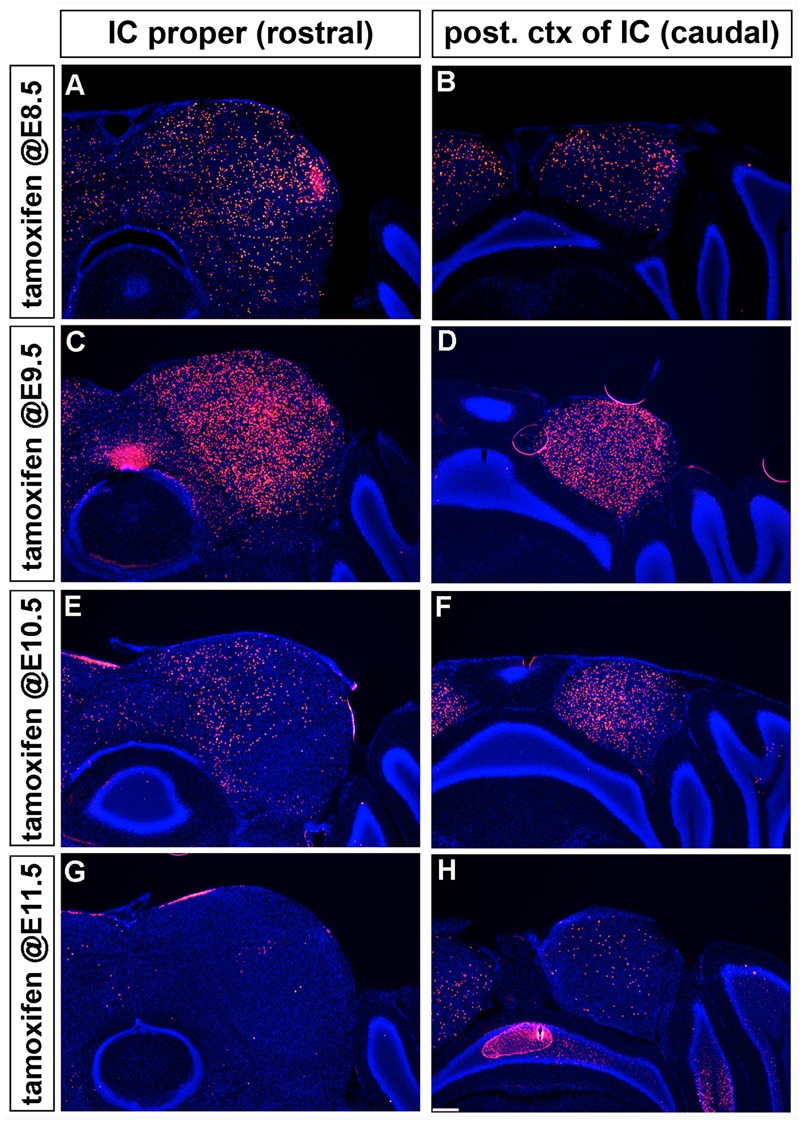
Temporal contribution of the *Wnt1* lineage to the auditory midbrain. **(A–D)** *Wnt1*-expressing progenitors marked early (at E8.5 and E9.5) contributed to neurons throughout the D-V and A-P extent of the auditory midbrain. **(E,F)** *Wnt1*-expressing progenitors marked at E10.5 populated the medial portion of the inferior colliculus proper and the posterior region of the inferior colliculus, although less extensively than at E9.5. The drop off was more pronounced in the IC proper **(E)** than in the posterior IC **(F)**. **(G,H)** *Wnt1*-expressing progenitors marked at E11.5 ceased to contribute to the anterior inferior colliculus, but sparsely contribute to the posterior inferior colliculus.

**FIGURE 8:**
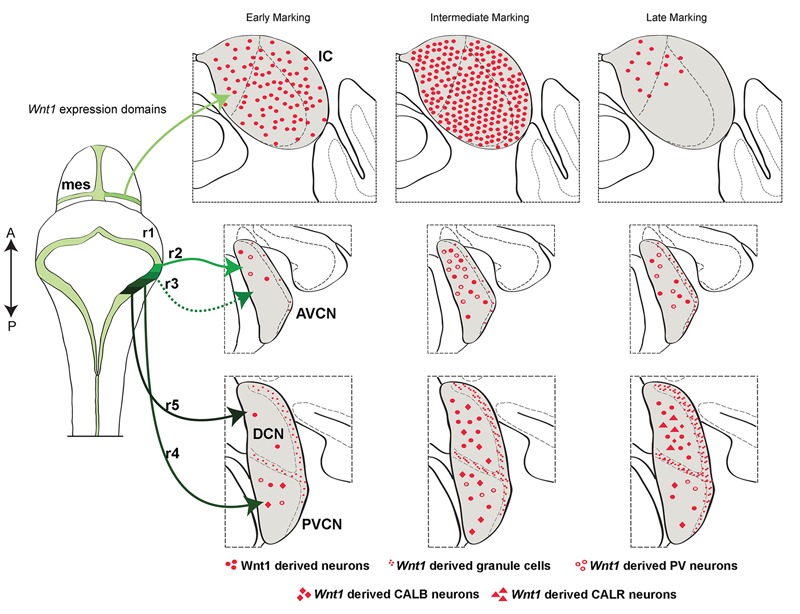
Summary schematic of the temporal allocation of *Wnt1*-expressing progenitors to mature midbrain and hindbrain auditory domains. A schematic of the dorsal view of the mouse mid-hindbrain during embryogenesis shows the *Wnt1* expression domains (green-black) and the contributions from the mesencephalon (mes) and rhombomeres (r) 2–5 to the mature inferior colliculus (IC) and cochlear nuclei. The IC was derived from *Wnt1*-expressing progenitors in the dorsal mes. Note: specific rhombomere contributions are based on Farago et al. (2006). Early, intermediate, and late marking is correlated to specific structures as indicted. **Light green lines** show the *Wnt1* lineage contribution to the auditory midbrain: Early (E8.5) marking of *Wnt1*-expressing progenitors in the mes had a diffuse contribution to the IC. Intermediate (E9.5) marking produced the peak contribution to the IC. Late (E10.5) marking conferred sparse contribution to dorsomedial aspects of the inferior colliculus. **Intermediate green lines** indicate *Wnt1* lineage contribution to auditory hindbrain cochlear nuclei: The AVCN is derived from *Wnt1*-expressing progenitors positioned in r2 and r3. Early marking (E8.5) of *Wnt1*-expressing progenitors resulted in sparse contribution to PV+ neurons (open circles) in the dorsal tier of the AVCN. Intermediate marking (E10.5) revealed greater contribution to PV+ neurons, while late (E12.5) marking resulted in a decrease of *Wnt1*-derived neurons in the AVCN. **Black lines** show the DCN is derived from *Wnt1*-expressing progenitors located in r5. Early marking (E8.5) of *Wnt1*-expressing progenitors revealed a primary contribution to granule cells of the MNS (small red dots). Magnocellular neurons of the DCN were derived from progenitors expressing *Wnt1* at an intermediate stage (E10.5), including CALB-expressing neurons (red diamonds). CALR-expressing neurons (red triangles) were derived from late (E12.5) *Wnt1* expression. **Dark green line** shows that the PVCN is derived from *Wnt1*-expressing progenitors of r4. The distribution of neurons derived from *Wnt1*-expressing progenitors resembles that of the AVCN, though PV+ and CALB+ neurons were derived from each stage examined.

Marking the *Wnt1* lineage at E8.5 or E9.5 resulted in cells being distributed along the rostral-caudal axis of the inferior colliculus with the most substantial contribution occurring at E9.5 (**Figures 7A–D**). The distribution of the *Wnt1* lineage marked at E10.5 was largely confined to the dorsomedial aspects of the anterior (rostral) inferior colliculus while the most significant contribution occurred in the posterior domain of the inferior colliculus (**Figures 7E,F**). In contrast, marking at E11.5 did not yield appreciable contribution to the inferior colliculus proper anteriorly and resulted in a significant decline in contribution to the posterior inferior colliculus (**Figures 7G,H**). Collectively, GIFM revealed the differential contribution of the *Wnt1* lineage marked at specific time points (described above) to the auditory hindbrain and midbrain, which is summarized in **Figure 8**.

### *Wnt1* Is Functionally Required for Establishing Hindbrain and Midbrain Auditory Centers

**FIGURE 9:**
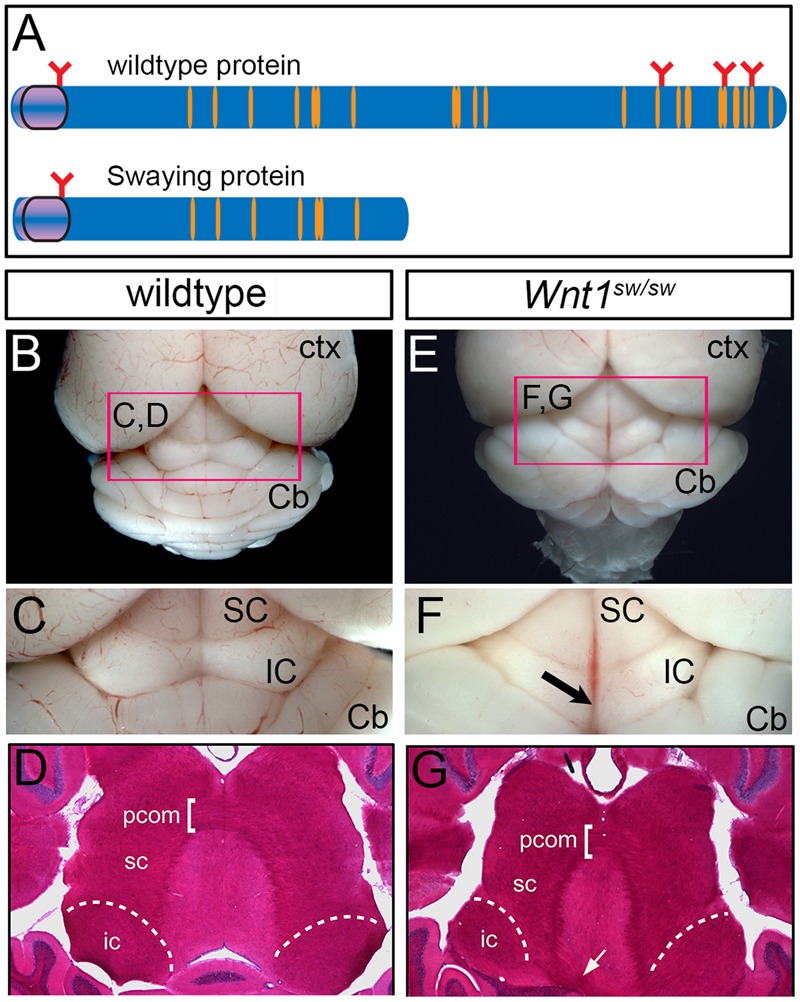
Whole mount view and horizontal sections showing adult brain structures of wild type and *Wnt1^sw/sw^* mice. **(A)** Schematic of wild type WNT1 and Swaying mutant proteins; orange indicates cysteine residues, red indicates glycosylation sites. **(B)** Wild type whole-mount photomicrograph showing the caudal aspect of the cerebral cortex (Ctx) and cerebellum (Cb) at low magnification. The dorsal midbrain is delineated by the pink marque. **(C)** The superior colliculus (SC) and inferior colliculus (IC) of a representative wild-type mouse is shown at higher magnification. **(D)** A horizontal section stained with hematoxylin and eosin (H&E) shows the dorsal midbrain cytoarchitecture of a control wild-type mouse including the posterior commissure (pcom). **(E)** Whole-mount photomicrograph of a representative *Wnt1^sw/sw^* mouse showing the caudal aspect of the cerebral cortex (Ctx) and the cerebellum (Cb) with midline/vermis defect at low magnification. The dorsal midbrain is perturbed as compared to wild-type mouse. **(F)** In particular, the IC is misshaped and has a lack of continuity at the midline (arrow). **(G)** A horizontal section of a *Wnt1^SW/SW^* mouse stained with H&E shows aberrant fiber tracts emanating from adjacent to the inferior colliculus (white arrow).

We took advantage of mice homozygous for the *Swaying* allele *(Wnt1^sw/sw^)* to investigate how a mutation in *Wnt1* alters the development of the cochlear nucleus (hindbrain) and the inferior colliculus (midbrain). The *swaying* allele is a naturally occurring single base pair deletion in the coding sequence of *Wnt1* that is believed to result in truncated WNT1 protein with hypomorphic function (Thomas et al., 1991). *Wnt1^sw/sw^* mice display variable gross morphological phenotypes of the midbrain and cerebellum (Thomas et al., 1991; Ellisor et al., 2012) (**Figure 9**).

**FIGURE 10:**
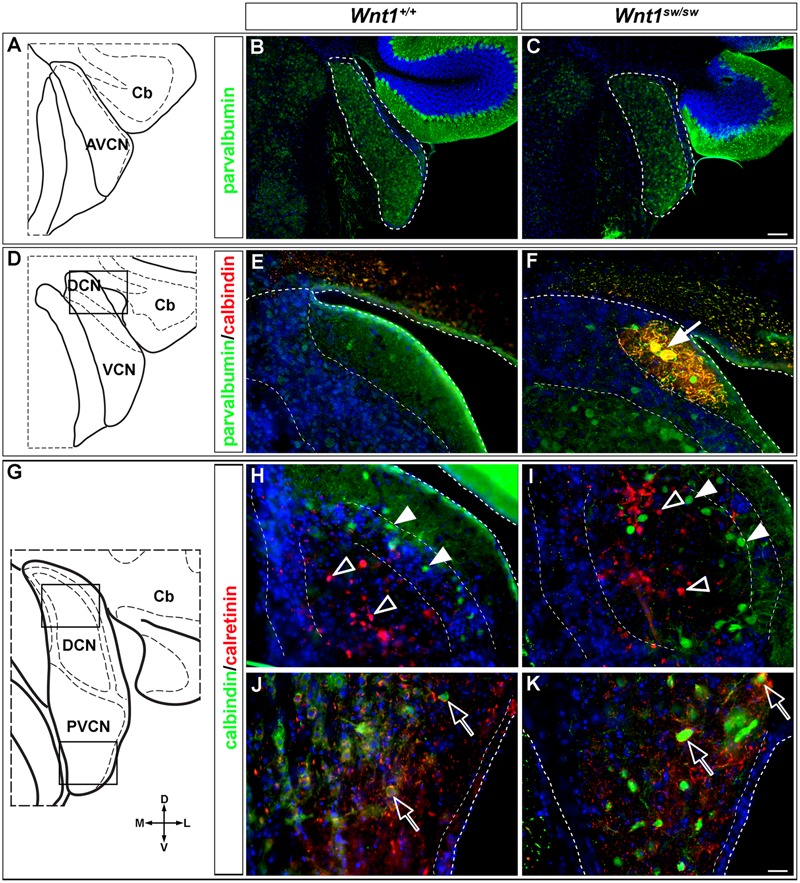
Altered morphology and ectopic cell types in the cochlear nucleus of *Wnt1^sw/sw^* mice. **(A)** Schematic representation of the AVCN. **(B)** Normal morphology observed in control AVCN. **(C)** Stereotypical and broadened morphology observed in the AVCN of *Wnt1^sw/sw^* mice. **(B,C)** PV-expressing neurons of the AVCN were prevalent in controls and mutants. **(D)** Schematic view of the DCN/VCN in sectioning planes and boxed areas analyzed in **(E,F)**, depicting the transition between AVCN and PVCN (denoted VCN) and the most anterior aspects of the DCN. While the general morphology of mutant posterior cochlear nuclei remained intact, an ectopic cell type characteristic of Purkinje-like cells (PLCs, white arrow) based on morphology and co-expression of PV/CALB) was commonly observed **(F)** in mutants versus controls **(E)**. **(G)** Schematic view delineating the boxed areas of the DCN and PVCN analyzed in **(H–K)**. **(H,I)** The general morphology of the DCN was largely normal in controls versus mutants. CALB (solid arrowheads) and CALR (open arrowheads) expressing neurons were generally unaffected although some CALB+ neurons were inappropriately localized to the core. The morphology and cell type specification appeared to be unaffected in the mutant PVCN. Open arrows highlight CALB+ neurons surrounded by CALR+ positive processes. Scale bars: 130 μm **(B,C)**; 32 μm **(E,F,H,I,J,K)**.

**FIGURE 11:**
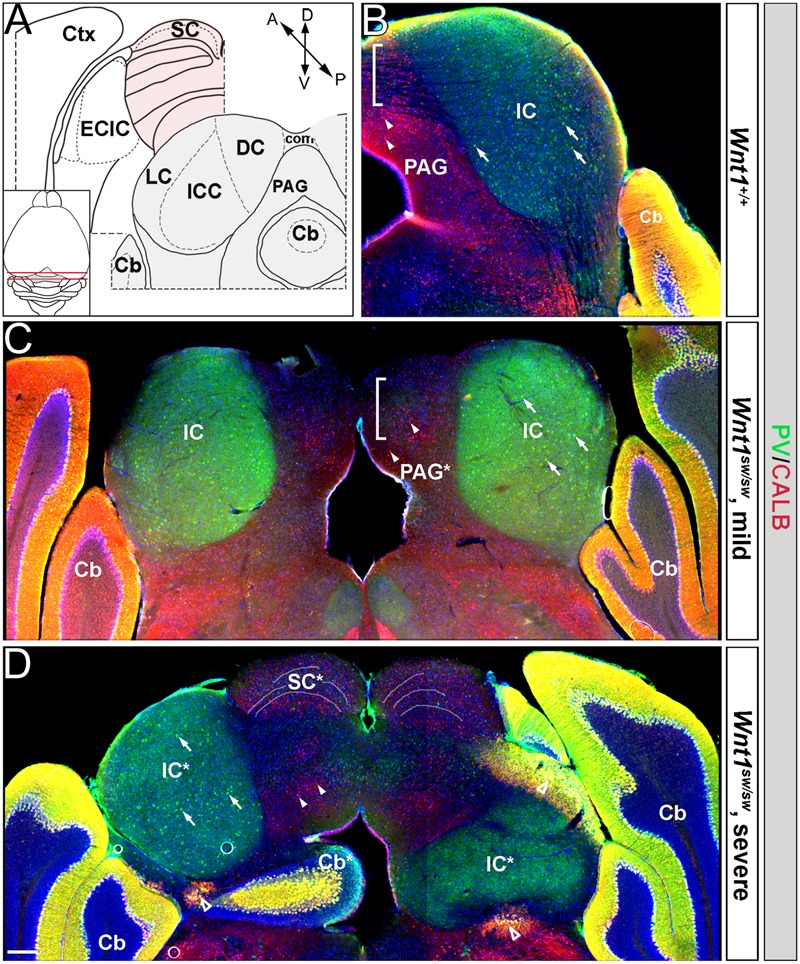
Patterning deficits of the auditory midbrain of *Wnt1^sw/sw^* mice. **(A)** Schematic representations of wild type hemi-coronal planes of section through the anterior and posterior dorsal midbrain. The posterior midbrain is analyzed in **(B–D)**. Inset: schematic of a dorsal whole mount brain depicts the anterior (pink shading) and posterior (gray shading) midbrain. The anterior diagram shows the superior colliculus (SC, highlighted in pink) and the external cortex of the inferior colliculus (ECIC, highlighted in gray). Laterally, the cortex (Ctx) surrounds the Mb in this plane. The posterior plane delineates each subdivision of the inferior colliculus proper: central nucleus (ICC), dorsal cortex (DC), and lateral cortex (LC). The periaqueductal grey (PAG) borders the inferior colliculus ventromedially. The cerebellum is denoted (Cb). **(B)** Hemi-coronal wild type section representative of the posterior plane shows normal staining of PV (green) and CALB (red). PV is broadly seen throughout the inferior colliculus (IC) while CALB is largely confined to the PAG. Examples of PV and CALB positive neurons are noted with arrows and arrowheads, respectively. Commissural connections between the colliculi are indicated by the bracketed area and by (com) in **(A)**. Phenotypes varied from mild **(C)** to severe **(D)** in *Wnt1^sw/sw^* mice. **(C)** *Wnt1^sw/sw^* mice with a mild phenotype exhibited minor changes in bilateral symmetry while the general morphology of each IC was maintained. Although the PAG was malformed (denoted by PAG^∗^) both PV and CALB neurons were specified. Commissural connections seen as black striations in wild-type mice **(B)** were absent in the bracketed domain **(D)**. *Wnt1^sw/sw^* mice with a severe phenotype displayed a loss of bilateral symmetry between each IC (as determined by PV expression and denoted as IC^∗^) The severe phenotype also had ectopic cerebellar tissue and cell types within the midbrain. Finally, there was the presence of presumptive SC tissue that was positioned anteriorly in controls (see illustration in A). The left IC^∗^ in **(D)** was established with apparently normal morphology and contained PV-expressing neurons, while the right IC^∗^ was severely perturbed and contained few PV-expressing neurons. Ectopic or malformed Cb tissue was present within the Mb (Cb^∗^), which contained ectopic Purkinje cells that expressed both PV and CALB (open arrowheads). Presumptive anterior tissue (SC^∗^) was inappropriately positioned and was observed in the same plane as the IC (compare **B** and **D**), which was evident by the dorsal laminated morphology and expression of CALB. Scale bar: 260 μm **(B–D)**.

**FIGURE 12:**
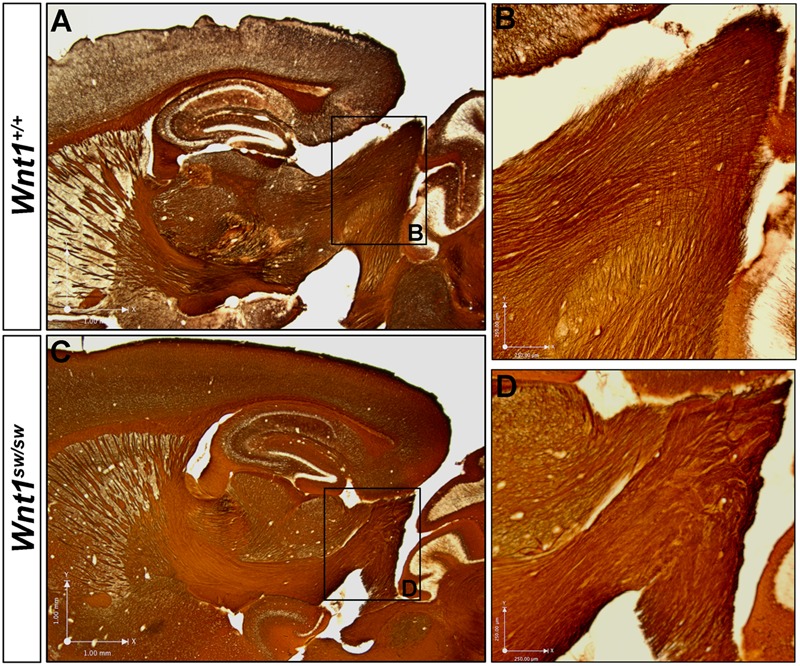
*Wnt1* is required for normal axonal organization in the brachium of the inferior colliculus. Myelin-based silver staining allowed for the visualization of axons leaving the inferior colliculus *en route* to higher order auditory centers. **(A,B)** Wild type axons apparently exiting the auditory midbrain formed organized, parallel fibers. **(C,D)** *Wnt1^sw/sw^* mice displayed clumped, disoriented axonal bundles. See **Figure 9** for morphological changes in the inferior colliculus and ectopic white matter fibers in horizontal orientation.

However, the degree to which the cell types of the auditory midbrain and hindbrain are affected in *Wnt1^sw/sw^* mice has not been reported. Therefore, we analyzed mutant versus control mice using cell type specific markers. Compared to controls, the anterior aspect of the cochlear nucleus was altered in *Wnt1^sw/sw^* mice (**Figures 10A–C**). Specifically, the AVCN was shorter along the D-V axis and broader along the M-L axis, which resulted in a subtle change in morphology (**Figures 10B,C**). We also observed the presence of large, ectopic Purkinje-like cells (PLC) in the anterior-dorsal aspects of the *Wnt1^sw/sw^* DCN (**Figures 10D–F**, arrow). In contrast, the gross morphology of the PVCN and posterior DCN were largely unaffected, although their were aberrant CALB-expressing neurons interspersed amongst CALR-expressing neurons in the DCN (**Figures 10G–K**). We also compared the inferior colliculus (schematic shown in **Figure 11A**) of *Wnt1^+/+^* and *Wnt1^sw/sw^* mice by immunolabeling for PV and CALB (**Figures 11B–D**). PV is a broad marker of the three inferior colliculus subdivisions, including the entire central nucleus (ICC) and aspects of the surrounding dorsal (DC) and lateral (LC) cortices (Coleman et al., 1992; Vater and Braun, 1994; Lohmann and Friauf, 1996; Sharma et al., 2009). CALB is present in the cortices surrounding the ICC, as well as the periaqueductal gray (PAG), located ventromedially (Coleman et al., 1992; Vater and Braun, 1994; Paxinos, 1999; Sharma et al., 2009). In the wild type auditory midbrain there was clear delineation between PV and CALB positive neurons (**Figure 11B**). Although much of the staining was diffuse due to PV and CALB positive afferents, the cell bodies of PV+ and CALB+ neurons were readily identified in *Wnt1^+/+^* mice (**Figure 11B**, arrows and arrowheads, respectively). In *Wnt1^sw/sw^* mice, which exhibited a mild phenotype, the medial component of the PAG was distorted (**Figure 10C**, PAG^∗^). In addition, the commissural connections between colliculi of each side were not easily distinguished (compare bracketed areas in **Figure 11B** vs. **Figure 11C**). However, both PV and CALB-expressing neurons were abundant and generally positioned in proper anatomical locations (**Figure 11C**, arrows and arrowheads, respectively). We observed *Wnt1^sw/sw^* mutants with a loss of bilateral symmetry, the presence of ectopic cell types, and aberrant A-P patterning in the midbrain, which we refer to as a severe phenotype (**Figure 11D**). Notably, the presumptive inferior colliculi were displaced along the D-V and A-P axes, and contained fewer PV-expressing cell bodies than controls (**Figure 11D**, IC^∗^, right side in this example). CALB-expressing neurons were easily identified (**Figure 11D**, arrowheads), but there was no clear delineation of the PAG, DC, or LC. The presumptive superior colliculus (SC^∗^) in *Wnt1^sw/sw^* mutants, based on morphology and laminated expression of CALB, was found in plane with the presumptive ICC (**Figure 11D**). Ectopic cerebellar Purkinje cell types as determined by morphology and co-expression of PV/CALB were found in clusters throughout the midbrain (**Figure 11D**, open arrowheads). In addition to morphological and cellular differences, afferents coursing through the ICC delineated by silver staining in *Wnt1^sw/sw^* mice were disorganized as they passed through the brachium en route to higher order auditory centers (**Figures 12A,B** vs. **Figures 12C,D**). These results confirm that *Wnt1* is required for the normal specification of auditory midbrain neurons as well as the proper organization of commissural connections and afferents of the inferior colliculus.

## Discussion

We used *Wnt1-Venus* embryos to validate that *Wnt1* expression dynamically changes at early stages and rapidly becomes fixed following neurulation. We then used GIFM to quantify the contribution of *Wnt1-*expressing progenitors to the auditory hindbrain and midbrain. We also took advantage of GIFM to assess two distinct models of how *Wnt1*-expressing progenitors contribute to biochemically distinct neurons of the auditory hindbrain and midbrain. Specifically, we tested whether auditory structures as well as unique cell types were derived entirely from an early pool of *Wnt1*-expressing progenitors (progressive lineage restriction model) or whether waves of *Wnt1* expression (*de novo* expression model) are related to the competence of *Wnt1*-expressing progenitors that contribute to mature auditory structures. Interestingly, the *de novo* expression model appropriately explains the contribution to the hindbrain. In contrast, a mixed model dominated by progressive lineage restriction best explains the contribution to the midbrain. Thus, both mechanistic models are employed in the development of *Wnt1-*derived auditory structures. Finally, we examined the morphology and cell types of the cochlear nucleus and inferior colliculus in *Wnt1^sw/sw^* mice and show minor deficits in the cochlear nucleus, but more severe perturbations in the inferior colliculus, which suggests that perturbing the lineage restriction model has a more dramatic impact on auditory brain structures.

### *Wnt1* Lineage Contribution to the Auditory Hindbrain

We utilized GIFM to characterize the profile and distribution of neurons derived from the *Wnt1* lineage and showed that the *Wnt1* lineage contributes to the cochlear nucleus in complex waves of *de novo Wnt1* expression. This model is nicely illustrated by assessing the contribution to the DCN. Notably, if the DCN was derived entirely from progenitors expressing *Wnt1* early, we would have expected a contribution to all cell types including granule cells and magnocellular neurons. However, we observed that the MNS (granule neurons) is derived from *Wnt1*-expressing progenitors throughout the span of *Wnt1* expression (E8.5–E12.5), but the large projecting neurons of the magnocellular core are generated after E10.5. Notably, the competence state of progenitors to give rise to biochemically distinct cell types does not diminish as development proceeds. Therefore, *de novo* expression of *Wnt1* in auditory progenitors occurs within the LRL after E10.5 to generate CALB+ and CALR+ neurons of the DCN. We quantified the contribution of *Wnt1*-expressing progenitors to cochlear nuclei over time and show that *de novo* peaks of *Wnt1* expression occurred. These results show that despite broad expression of *Wnt1* throughout the LRL germinal zone, progenitors exist that do not express *Wnt1* early, but turn it on at later developmental stages.

### *Wnt1* Lineage Contribution to the Auditory Midbrain

The inferior colliculus of the midbrain is a complex structure and harbors neurons that express periodicity proteins, which regulate a circuidian clock (Park et al., 2016). In addition, the inferior colliculus is innervated by dopamine neurons of the subparafascicular thalamic nucleus (Nevue et al., 2016). Our fate mapping results clarify how neurons are established and contribute to the mature auditory midbrain. Specifically, we show that the inferior colliculus is not entirely derived from progenitors that express *Wnt1* early, which is consistent with the presence of both *Wnt1*-expressing and non-expressing progenitors that are intermingled within the mesencephalon at E8.5 (**Figure 1**). Thus, *Wnt1*-expressing progenitors marked at E8.5 results in a relatively diffuse contribution through the extent of the inferior colliculus (**Figure 8**). *Wnt1* expression in the mesencephalon dynamically changed, coinciding with the closure of the anterior neural fold (at E9.5). Notably, the peak contribution to inferior colliculus neurons arose from *Wnt1*-expressing progenitors marked at E9.5. These findings imply that *de novo Wnt1* expression occurred in the dorsal mesencephalon over the span of 24 h. Subsequently, the contribution of *Wnt1*-expressing progenitors to the inferior colliculus rapidly decreased (with marking at E10.5). A model of progressive lineage restriction best describes how progenitors contribute to the inferior colliculus following neural tube closure. Collectively, our results show that the auditory midbrain is established by a mixed model of *Wnt1* lineage-contribution in which an early wave of *de novo* expression is replaced by a progenitor population that is progressively restricted over time.

### GIFM of the *Wnt1* Lineage versus Birth Dating in Auditory Structures

In the current study we used a Swiss Webster (mouse) background for lineage mapping. However, studies using ^3^H-thymidine labeling for birth dating of neurons were typically conducted in rat (Altman and Bayer, 1981). These classic studies have been the gold standard of birth dating. Thus, we converted rat to mouse embryonic stages of development (Supplementary Table 1) to contrast our GIFM with birth dating studies. Using this approach for comparison indicates there is contribution of the *Wnt1* lineage at E8.5_mouse_ (E9.6_rat_) with the peak of contribution along the rostral-caudal axis occurring at E9.5_mouse_ (E10.8_rat_) with a significant decrease at E10.5_mouse_ (E12.0_rat_). The lateral aspect of the inferior colliculus has a peak of birth dating between E16 and E17_rat_ which is estimated to be post-E12.5_mouse_. However, at E12.5_mouse_ the *Wnt1*-lineage no longer contributes the inferior colliculus. Similarly, the medial aspect of the inferior colliculus has a peak of birth dating between E17 and E19_rat_ which is estimated to be E15.5–E16.6_mouse_. Notably, this time period is also later than *Wnt1* expression and lineage contribution in the inferior colliculus. Finally, a similar trend is observed in the DCN of the hindbrain: peak birth dating of the DCN is E14–E16_rat_ (E12–E14_mouse_) (Altman and Bayer, 1980). Thus, birth dating occurs subsequent to *Wnt1* lineage allocation, which happens between E10.5–E12.5 in mouse. In summary, *Wnt1* is extinguished prior to terminal proliferation and suggests that genetic lineage is established in the inferior colliculus and DCN of the hindbrain earlier than what might be suggested by birth dating studies. Interestingly, a similar observation was found when using GIFM to determine *Gli1* lineage allocation during dopamine neuron development (Hayes et al., 2011). However, this is not a universal theme as *Wnt1* lineage allocation occurs concomitantly with birth dating in the cerebellum and pre-cerebellar system (Hagan and Zervas, 2012). More broadly and in the context of the current study, these comparisons suggest that the *Wnt1* lineage is established prior to birth dating in the auditory midbrain and hindbrain.

### *Wnt1* Is Required for the Development of Central Auditory Structures

Notably, *Wnt1^sw/sw^* mice have a truncated and broadened AVCN and the presence of ectopic PLCs in the anterior DCN (**Figure 10**). The presence of these cells, which have been referred to as displaced Purkinje cells or PLCs, has been observed in the brainstem of wild type rats, including in the cochlear nucleus (Hurd and Feldman, 1994; Koszeghy et al., 2009). In comparison, the location of PLCs within the DCN of *Wnt1^sw/sw^* mice suggests that the expression of *Wnt1* normally prevents their presence or specification within this region in mice. *Wnt1* is also required for the development of the auditory midbrain. In both mildly and severely affected *Wnt1^sw/sw^* mice, the central nucleus and external cortex of the inferior colliculus were present. However, the auditory midbrain in severely affected mice was aberrantly patterned. Severe *Wnt1^sw/sw^* mutants had ectopic anterior midbrain and misplaced cerebellar tissues surrounding the presumptive inferior colliculus. In one example, half of the auditory midbrain was largely void of PV+ cell bodies, and commissural connections between each Inferior colliculi could not be detected. In addition, axons of the IC were greatly disorganized. It is known that the loss of *Wnt1* perturbs the formation of the isthmus organizer required for patterning the adjacent midbrain and cerebellum (Adams et al., 2000; Matsunaga et al., 2002; Ellisor et al., 2012), yet how this perturbation affected the development of the inferior colliculus and its subdivisions had not been determined. While it is likely that much of the patterning deficits we observe are secondary to the disruption of the isthmus organizer, *Wnt1* is apparently also required for the proper specification of auditory structures and their connections.

One prominent deficit common to both mildly and severely affected *Wnt1^sw/sw^* mice is the loss or displacement of commissural connections between the colliculi. Moreover, the axonal output of the inferior colliculus was greatly disorganized in mildly affected mice. Late *Wnt1* expression may therefore be instructive in establishing inter-collicular projections or to promote the outgrowth of anterior projections *en route* to the medial geniculate body in the thalamus. Lastly, the presence of a late WNT1 signal in the mesencephalon may serve as an attractant or remodeling cue for ascending input from developing hindbrain auditory centers. Projections from the cochlear nucleus reach the midbrain as early as E15 in rat, which is on par with late *Wnt1* expression in the dorsal mesencephalon (note: E15 in rat roughly corresponds to E13 in mouse (Clancy et al., 2001 and Supplementary Table 1). This suggests a mechanism of molecular matching where auditory neurons of temporally defined lineages may be functionally connected by axonal projections.

### *Wnt1* Lineage Contribution to Auditory Structures versus *Wnt1* Mutant Phenotypes

Although we demonstrated that *Wnt1* is required for proper development of the hindbrain cochlear nucleus and the midbrain inferior colliculus using mice with a perturbation in *Wnt1*, it may appear that there is discordance between our fate mapping results and the *Wnt1^sw/sw^* phenotype. However, subtle differences between fate mapping results and the *Wnt1^sw/sw^* phenotype could be due to the fact that the population of *Wnt1*-expressing cells (marked by GIFM) and the population of cells that respond to *Wnt1* signaling are not identical and might only partially overlap. Additionally, GIFM results in a mosaic tapestry of cells marked within a distinct temporal window, while the *Wnt1^sw/sw^* line is the result of a germ line mutation, which may contribute to differences. Finally, it cannot be fully excluded that *Wnt1-CreER^T^* expression differs (slightly) from endogenous *Wnt1* expression. However, it can be construed that divergent expression of *Wnt1-CreER^T^* and *Wnt1* is unlikely since our previously published analysis of the initial marked populations using this line did not provide evidence that the two expression patterns differ from each other (Ellisor et al., 2009; Hagan and Zervas, 2012).

### Potential Roles of *Wnt1* in Auditory System Development

The *Wnt1* lineage which gives rise to the hindbrain cochlear nucleus is not progressively restricted over time. We show that waves of *de novo Wnt1* expression are differentially correlated with the distribution and emergence of unique cell types, which suggests that *Wnt1* may not play a primary role in promoting the proliferation of cochlear nucleus progenitors. Rather, our results suggest temporal roles for *Wnt1* in specifying distinct cell types within the cochlear nucleus subdivisions. A notable finding is that *Wnt1* contributes to functional classes of auditory hindbrain neurons. While we hypothesized that biochemically distinct neurons (expressing PV, CALB, or CALR) would be differentially derived based on the timing of *Wnt1* expression, this was true only for CALB and CALR neurons of the DCN. Interestingly, the biochemical signature in each case correlates with neurons that form contralateral excitatory circuits (Fredrich et al., 2009). This could represent a mechanisms where lineage allocation and neural circuits are coupled. The *Wnt1* lineage primarily established excitatory cell types such as PV-expressing neurons of the AVCN/PVCN. These cells are thought to be T-stellate/multipolar type I neurons that provide excitatory contralateral innervation of the inferior colliculus (Cant and Benson, 2003).

Outstanding questions are what are the downstream molecular targets of *Wnt1* and how might they help decipher a pathway used in development? Through cumulative genetic fate mapping, Fujiyama demonstrated that progenitors that express the bHLH transcription factors *Math1* and *Ptf1a* give rise to excitatory and inhibitory cell types of the cochlear nucleus, respectively. *Math1* is contained within the dorsal tier of *Wnt1* expression in the LRL (Landsberg et al., 2005), while *Ptf1a* is believed to be expressed just ventral to or slightly overlapping with the weakest ventral domain of *Wnt1* expression (Yamada et al., 2007; Fujiyama et al., 2009). Differing levels of WNT1 may regulate the expression of these transcription factors, such that high levels promote *Math1*, while low levels promote *Ptf1a*. Of importance is determining whether these genes are regulated by canonical Wnt signaling, as demonstrated for neural crest lineages of the developing hindbrain (Lee et al., 2004). Regardless, our fate mapping studies provide a comprehensive assessment of the how temporal expression of *Wnt1* is related to auditory lineage allocation and we provide functional evidence for the role of *Wnt1* in the development of the auditory hindbrain and midbrain.

## Ethics Statement

Mice were housed and handled in accordance with Brown University Institutional Animal Care and Use Committee (IACUC) guidelines (Genetic Approaches Using *Mus Musculus* as a Model Organism to Understand Mechanisms Underpinning Neurodevelopment and Neurological Disorders in Vivo”, IACUC #1209030).

## Author Contributions

The original experimental approach was designed by SB and MZ. Experiments were conducted by SB. The manuscript was written and edited by SB and MZ. Research presented here fulfilled in part the Ph.D. thesis requirement for SB.

## Conflict of Interest Statement

The authors declare that the research was conducted in the absence of any commercial or financial relationships that could be construed as a potential conflict of interest.
