# Supplementary material for: Temporal Expression of Wnt1 Defines the Competency State and Terminal Identity of Progenitors in the Developing Cochlear Nucleus and Inferior Colliculus
Source: Front Neuroanat. 2017 Aug 22;11:67. doi: 10.3389/fnana.2017.00067 (PMC5572273; doi:10.3389/fnana.2017.00067)
Supplement: Supplementary file 1 [file Table_1.pdf]

Developmental Stages of Comparison		
Rat (Purdue-Wistar)	Mouse (Swiss Webster)	
Actual	Approximate	Converted
E9.6 (approx.)	E8.5	E8.5
E10.8 (approx.)	E9.5	E9.5
E11	E10.0	E9.75
E12	E10.5	E10.5
E13	E11.5	E11.5
E14	E12.0	E12.3
E15	E13.0	E13.2
E16	E14.0	E14.1
E17	E15.0	E15.0
E18	E16.0	E15.9
E19	E16.5	E16.7
E20	E17.5	E17.6
E21	E18.5	E18.5

Supplemental Table 1. Comparison of developmental stages in rats and mice. The gestational period of Purdue-Wistar rats is 21d (Altman and Bayer, 1981). In contrast, Swiss Webster mice used for lineage mapping in this study have a gestational period of 18.5 days (based on breeding Swiss Webster Mice in Zervas lab over seven years (Approximate). Thus, there is a 1.135d difference between Purdue-Wistar rats and Swiss Webster mice. We used 1.135 to convert rat gestational stages to mouse stages (Converted) and then rounded to the nearest 0.5 day to generate an approximate equivalent age in mice. The conversion is used in the discussion of the manuscript to compare stages of development in classic birthdating and our fate mapping results.
